# Triaqua­bis{μ-*N*-[*N*-(4-meth­oxy-2-oxidobenzyl­idene)glyc­yl]glycinato(3−)}cadmium(II)dicopper(II) dihydrate

**DOI:** 10.1107/S1600536809039440

**Published:** 2009-10-03

**Authors:** Jiaxun Jiang, Yao Lu, Limin Yuan, Wenlong Liu

**Affiliations:** a College of Chemistry and Chemical Engineering, Yangzhou Universitry, Yangzhou 225002, People’s Republic of China

## Abstract

In the title compound, [CdCu_2_(C_12_H_11_N_2_O_5_)_2_(H_2_O)_3_]·2H_2_O, the Cu^II^ atoms are in a square plane of N_2_O_2_ atoms contributed by the tetra­dentate Schiff base trianion. The Cu^II^ atoms are coordinated by one phenolate O atom, one imine N atom, one amido N atom and one carboxyl­ate O atom. The Cd^II^ atom is connected *via* the carboxyl­ate groups, forming a heterotrinuclear Cu^II^–Cd^II^–Cu^II^ system. The Cd^II^ atom is seven-coordinate in a penta­gonal-bipyramidal geometry with four O atoms from two carboxyl­ate groups and three aqua ligands. The heterotrinuclear mol­ecules are linked to the uncoordinated water mol­ecules by O—H⋯O hydrogen bonds into a three-dimensional framework.

## Related literature

For the magnetic properties of heteronuclear Schiff-base complexes, see: Liu *et al.* (2004 ▶); Zou *et al.* (2003 ▶); Wu *et al.* (2007 ▶); Costes *et al.* (2006 ▶). For their optical properties; see: Akine *et al.* (2008 ▶). For the synthesis, see: Miyasaka *et al.* (1996 ▶).
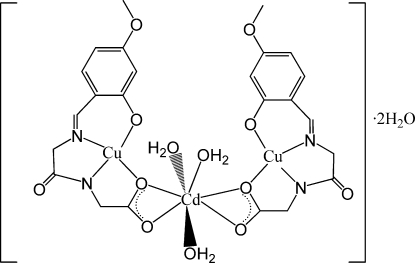


## Experimental

### 

#### Crystal data

[CdCu_2_(C_12_H_11_N_2_O_5_)_2_(H_2_O)_3_]·2H_2_O
*M* *_r_* = 856.02
Triclinic, 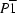

*a* = 9.813 (2) Å
*b* = 12.547 (3) Å
*c* = 12.598 (3) Å
α = 94.175 (4)°
β = 103.168 (3)°
γ = 90.148 (4)°
*V* = 1506.0 (6) Å^3^
*Z* = 2
Mo *K*α radiation
μ = 2.18 mm^−1^
*T* = 296 K
0.30 × 0.25 × 0.22 mm

#### Data collection

Bruker SMART APEX CCD diffractometer
Absorption correction: multi-scan (*SADABS*; Sheldrick, 2004 ▶) *T*_min_ = 0.529, *T*_max_ = 0.619
7383 measured reflections
5169 independent reflections
4763 reflections with *I* > 2σ(*I*)
*R*_int_ = 0.072

#### Refinement

*R*[*F* ^2^ > 2σ(*F* ^2^)] = 0.041
*wR*(*F* ^2^) = 0.110
*S* = 1.05
5169 reflections
417 parameters
H-atom parameters constrained
Δρ_max_ = 1.00 e Å^−3^
Δρ_min_ = −1.13 e Å^−3^

### 

Data collection: *SMART* (Bruker, 2002 ▶); cell refinement: *SAINT-Plus* (Bruker, 2003 ▶); data reduction: *SAINT-Plus*; program(s) used to solve structure: *SHELXS97* (Sheldrick, 2008 ▶); program(s) used to refine structure: *SHELXL97* (Sheldrick 2008 ▶); molecular graphics: *SHELXTL* (Sheldrick, 2008 ▶) and *DIAMOND* (Brandenburg, 2006 ▶); software used to prepare material for publication: *SHELXTL*.

## Supplementary Material

Crystal structure: contains datablocks I, global. DOI: 10.1107/S1600536809039440/ng2653sup1.cif

Structure factors: contains datablocks I. DOI: 10.1107/S1600536809039440/ng2653Isup2.hkl

Additional supplementary materials:  crystallographic information; 3D view; checkCIF report

## Figures and Tables

**Table 1:** Hydrogen-bond geometry (Å, °)

*D*—H⋯*A*	*D*—H	H⋯*A*	*D*⋯*A*	*D*—H⋯*A*
O11—H11*D*⋯O14	0.85	1.91	2.715 (4)	158
O13—H13*A*⋯O6	0.85	2.14	2.987 (4)	175
O13—H13*C*⋯O1	0.85	2.21	2.829 (4)	129
O14—H14*B*⋯O9	0.85	2.48	3.045 (4)	125
O11—H11*C*⋯O7^i^	0.85	1.91	2.722 (4)	159
O12—H12*D*⋯O2^ii^	0.85	2.11	2.667 (4)	123
O12—H12*E*⋯O15^iii^	0.85	2.13	2.737 (4)	129
O14—H14*A*⋯O7^iv^	0.85	2.17	2.791 (4)	130
O15—H15*B*⋯O4^v^	0.85	2.55	3.067 (4)	121
O15—H15*C*⋯O9^iv^	0.85	2.36	2.934 (4)	126

Symmetry codes: (i) 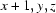; (ii) 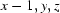; (iii) 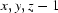; (iv) 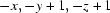; (v) 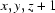.

**Fig. 1.**
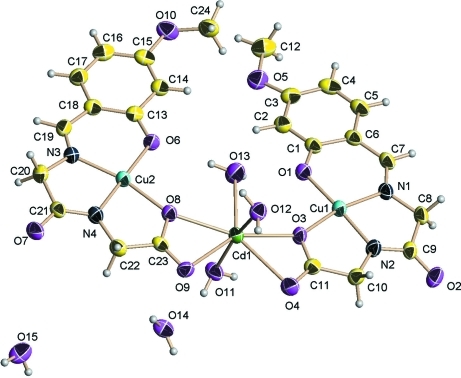
The molecular structure of (I), with atom labels and 50% probability displacement ellipsoids.

**Fig. 2.**
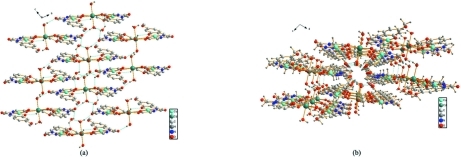
(a) A schematic representation of the two-dimensional structure formed via hydrogen bonds, viewed from the ac-plane, showing the hydrogen bond hexagon rings. (b) A packing diagram for (I), viewed down the b-axis, showing a three-dimensional framework connected by O—H···O hydrogen bonds (dashed lines).

## supplementary crystallographic information

### Comment

In recent years, the design and synthesis of Schiff base heteronuclear complexes that provide catalyst, biological activity, optical, magnetic materials (Wu *et al.*, 2007; Costes *et al.*, 2006; Liu *et al.*, 2004; Zou *et al.*, 2003; Akine *et al.*, 2008) caused an increasing interest in coordination chemistry. One of the best strategies to design heterometallic species is the 'complex as ligand' approach (Miyasaka *et al.*, 1996). In this article, we present the synthesis and structure of the title heterotrinuclear Schiff base complex derived from 4-methoxy-salicylaldehyde and glycylglycine.

The complex (I) is a asymmetric trinuclear [(CuL)_2_Cd(H_2_O)_3_] unit with the the *L*^3-^ bound to Cu^II^ and Cd^II^ atoms and crystallizes in the triclinic space group *P*1 (Fig. 1). Two [CuL]^-^ groups are connected by Cd^II^ cation in *cis* form to constitute a trinuclear Cu^II^–Cd^II^–Cu^II^ unit with Cu···Cu distances of 7.541 Å. [CuL]^-^ anions have approximately square-planar structures. The Schiff base ligand *L*^3-^ acts as a triple negatively charged quadridentate ONNO chelate and coordinated to the Cu^II^ atom *via* one phenolic oxygen, one imino nitrogen atom, one deprotonated amide nitrogen atom and one carboxylato oxygen atom. The Cu—O and Cu—N bond distances are in the range of 1.882 (2)–1.985 (2) Å and 1.889 (3)–1.923 (3) Å, respectively. The phenyl ring [C(1)—C(6)] / [C(13)—C(18)] and the chelate ring [O(1), C(1), C(6), C(7), N(1), Cu(1)] / [O(6), C(13), C(18), C(19), N(3), Cu(2)] make a small dihedral angle of 7.5 (2) ° / 8.9 (2) °, suggesting a large π-electron delocalization. The chelate rings [O(1), C(1), C(6), C(7), N(1), Cu(1)] and [O(6), C(13), C(18), C(19), N(3), Cu(2)] in the trinuclear moiety is almost parallel, with a small dihedral angle of 3.1 (2)°. The Cd^II^ atom is in a distorted pentagonal bipyramid environment, ligated by four carboxylato oxygen atoms (O(3), O(4), O(8), O(9)) arising from two [CuL]^-^ units and three aqua ligands (O(11), O(12), O(13)). The seven Cd—O bonds in the structure are in the range of 2.258 (3) - 2.545 (3) Å. In the crystal structure, the hydrogen bonds (Table 1), O(11)–O(14), O(7)–O(11) and O(7)–O(14) formed a hexagon ring (Fig. 2(*a*)). The hexagon rings are further connected by Cd^II^ ions and hydrogen bonds composing two-dimensional framework in *ac*-plane. The two-dimensional frameworks are further connected *via* the intermolecular hydrogen bonds O(12)–O(2), O(12)–O(15) and O(15)–O(9) to constitute a three-dimensional network (Fig. 2(*b*)).

### Experimental

Glycylglycine (5 mmol), 4-methoxy-salicylaldehyde (5 mmol) and LiOH (10 mmol) were dissolved in MeOH/H_2_O (30 ml, v:v = 1:1) and refluxed for 30 min. Then Cu(ClO_4_)_2_.6H_2_O (5 mmol) was added to the solution and the resulting solution was adjusted to 9–11 by 5 mol/*L* NaOH solution. After stirring at room temperature for 1 h, CdCl_2_.2.5H_2_O (2.5 mmol) was added. A violet precipitate was obtained immediately. After stirring for another 30 min and then filtrated, the precipitate was recrystallized from water. The violet crystals suitable for X-ray diffraction were obtained after one week. (yield 45% based on Cu(ClO_4_)_2_.6H_2_O).

### Refinement

The water H atoms in (I) were located in a difference Fourier map with a distance restraint of O—H = 0.85 Å and *U*_iso_(H) =1.5*U*_eq_(O). All other H atoms were positioned geometrically and constrained as riding atoms, with C—H distances of 0.93–0.97 Å and *U*_iso_(H) set to 1.2 or 1.5*U*_eq_(C) of the parent atom.

### Crystal data

**Table:**

[CdCu_2_(C_12_H_11_N_2_O_5_)_2_(H_2_O)_3_]·2H_2_O	*Z* = 2
*M**_r_* = 856.02	*F*(000) = 860
Triclinic, *P*1	*D*_x_ = 1.888 Mg m^−^^3^
Hall symbol: -P 1	Mo *K*α radiation, λ = 0.71073 Å
*a* = 9.813 (2) Å	Cell parameters from 6310 reflections
*b* = 12.547 (3) Å	θ = 2.4–27.8°
*c* = 12.598 (3) Å	µ = 2.18 mm^−^^1^
α = 94.175 (4)°	*T* = 296 K
β = 103.168 (3)°	Block, violet
γ = 90.148 (4)°	0.30 × 0.25 × 0.22 mm
*V* = 1506.0 (6) Å^3^	

### Data collection

**Table:**

Bruker SMART APEX CCD diffractometer	5169 independent reflections
Radiation source: fine-focus sealed tube	4763 reflections with *I* > 2σ(*I*)
graphite	*R*_int_ = 0.072
φ and ω scans	θ_max_ = 25.0°, θ_min_ = 2.1°
Absorption correction: multi-scan (*SADABS*; Sheldrick, 2004)	*h* = −11→11
*T*_min_ = 0.529, *T*_max_ = 0.619	*k* = −11→14
7383 measured reflections	*l* = −14→12

### Refinement

**Table:**

Refinement on *F*^2^	Primary atom site location: structure-invariant direct methods
Least-squares matrix: full	Secondary atom site location: difference Fourier map
*R*[*F*^2^ > 2σ(*F*^2^)] = 0.041	Hydrogen site location: inferred from neighbouring sites
*wR*(*F*^2^) = 0.110	H-atom parameters constrained
*S* = 1.05	*w* = 1/[σ^2^(*F*_o_^2^) + (0.0755*P*)^2^ + 0.7984*P*] where *P* = (*F*_o_^2^ + 2*F*_c_^2^)/3
5169 reflections	(Δ/σ)_max_ = 0.001
417 parameters	Δρ_max_ = 1.00 e Å^−^^3^
0 restraints	Δρ_min_ = −1.13 e Å^−^^3^

### Special details

**Table:**

Geometry. All e.s.d.'s (except the e.s.d. in the dihedral angle between two l.s. planes) are estimated using the full covariance matrix. The cell e.s.d.'s are taken into account individually in the estimation of e.s.d.'s in distances, angles and torsion angles; correlations between e.s.d.'s in cell parameters are only used when they are defined by crystal symmetry. An approximate (isotropic) treatment of cell e.s.d.'s is used for estimating e.s.d.'s involving l.s. planes.
Refinement. Refinement of *F*^2^ against ALL reflections. The weighted *R*-factor *wR* and goodness of fit *S* are based on *F*^2^, conventional *R*-factors *R* are based on *F*, with *F* set to zero for negative *F*^2^. The threshold expression of *F*^2^ > σ(*F*^2^) is used only for calculating *R*-factors(gt) *etc*. and is not relevant to the choice of reflections for refinement. *R*-factors based on *F*^2^ are statistically about twice as large as those based on *F*, and *R*- factors based on ALL data will be even larger.

### Fractional atomic coordinates and isotropic or equivalent isotropic displacement parameters (Å^2^)

**Table:**

	*x*	*y*	*z*	*U*_iso_*/*U*_eq_	
Cu1	0.54611 (4)	0.14993 (3)	0.11223 (4)	0.03071 (14)	
Cu2	−0.10904 (4)	0.15156 (3)	0.32980 (3)	0.02996 (14)	
Cd1	0.20503 (2)	0.285718 (19)	0.19598 (2)	0.03119 (12)	
C1	0.5331 (4)	−0.0578 (3)	0.1873 (3)	0.0314 (7)	
C2	0.4838 (4)	−0.1279 (3)	0.2518 (3)	0.0352 (8)	
H2	0.4140	−0.1058	0.2870	0.042*	
C3	0.5367 (4)	−0.2297 (3)	0.2644 (3)	0.0368 (8)	
C4	0.6384 (4)	−0.2668 (3)	0.2106 (3)	0.0399 (9)	
H4	0.6698	−0.3365	0.2156	0.048*	
C5	0.6902 (4)	−0.1979 (3)	0.1506 (3)	0.0379 (8)	
H5	0.7602	−0.2216	0.1162	0.046*	
C6	0.6440 (4)	−0.0933 (3)	0.1375 (3)	0.0318 (7)	
C12	0.5260 (5)	−0.3950 (3)	0.3460 (4)	0.0538 (11)	
H12A	0.5008	−0.4342	0.2758	0.081*	
H12B	0.4796	−0.4266	0.3957	0.081*	
H12C	0.6255	−0.3972	0.3736	0.081*	
C7	0.7203 (4)	−0.0258 (3)	0.0834 (3)	0.0340 (8)	
H7	0.7910	−0.0567	0.0540	0.041*	
C8	0.7864 (4)	0.1400 (3)	0.0230 (3)	0.0363 (8)	
H8A	0.7799	0.1131	−0.0522	0.044*	
H8B	0.8833	0.1364	0.0624	0.044*	
C9	0.7392 (4)	0.2552 (3)	0.0264 (3)	0.0309 (7)	
C10	0.5513 (4)	0.3681 (3)	0.0622 (3)	0.0351 (8)	
H10A	0.5175	0.3910	−0.0109	0.042*	
H10B	0.6151	0.4227	0.1048	0.042*	
C11	0.4297 (4)	0.3510 (3)	0.1151 (3)	0.0319 (8)	
C13	−0.0359 (4)	−0.0660 (3)	0.3442 (3)	0.0305 (7)	
C18	−0.1321 (4)	−0.0824 (3)	0.4113 (3)	0.0335 (8)	
C17	−0.1393 (4)	−0.1828 (3)	0.4521 (3)	0.0386 (8)	
H17	−0.1992	−0.1922	0.4984	0.046*	
C16	−0.0620 (4)	−0.2677 (3)	0.4267 (3)	0.0410 (9)	
H16	−0.0676	−0.3332	0.4559	0.049*	
C15	0.0253 (4)	−0.2527 (3)	0.3556 (3)	0.0371 (8)	
C14	0.0404 (4)	−0.1550 (3)	0.3168 (3)	0.0344 (8)	
H18	0.1021	−0.1472	0.2717	0.041*	
C24	0.1580 (5)	−0.3390 (3)	0.2383 (4)	0.0483 (10)	
H24A	0.0958	−0.3078	0.1789	0.072*	
H24B	0.1793	−0.4104	0.2152	0.072*	
H24C	0.2429	−0.2968	0.2601	0.072*	
C19	−0.2290 (4)	−0.0041 (3)	0.4348 (3)	0.0347 (8)	
H19	−0.2887	−0.0233	0.4782	0.042*	
C20	−0.3461 (4)	0.1646 (3)	0.4241 (3)	0.0378 (8)	
H20A	−0.3450	0.1684	0.5015	0.045*	
H20B	−0.4383	0.1395	0.3838	0.045*	
C21	−0.3162 (4)	0.2750 (3)	0.3911 (3)	0.0321 (7)	
C22	−0.1562 (4)	0.3748 (3)	0.3139 (3)	0.0363 (8)	
H22A	−0.1300	0.4271	0.3759	0.044*	
H22B	−0.2278	0.4051	0.2586	0.044*	
C23	−0.0309 (4)	0.3470 (3)	0.2686 (3)	0.0281 (7)	
N1	0.6983 (3)	0.0738 (2)	0.0726 (2)	0.0318 (6)	
N2	0.6222 (3)	0.2672 (2)	0.0577 (3)	0.0342 (7)	
N3	−0.2407 (3)	0.0901 (2)	0.4011 (2)	0.0339 (7)	
N4	−0.2090 (3)	0.2785 (2)	0.3474 (3)	0.0342 (7)	
O1	0.4761 (3)	0.03723 (19)	0.1772 (2)	0.0352 (6)	
O2	0.8121 (3)	0.3263 (2)	0.0003 (2)	0.0445 (7)	
O3	0.4089 (3)	0.2550 (2)	0.1407 (2)	0.0348 (6)	
O4	0.3531 (3)	0.4239 (2)	0.1324 (2)	0.0419 (6)	
O5	0.4843 (3)	−0.2862 (2)	0.3354 (2)	0.0467 (7)	
O6	−0.0138 (3)	0.02539 (18)	0.3055 (2)	0.0348 (6)	
O7	−0.3929 (3)	0.3516 (2)	0.4086 (2)	0.0435 (7)	
O8	0.0039 (3)	0.24752 (19)	0.2634 (2)	0.0327 (5)	
O9	0.0383 (3)	0.41583 (19)	0.2384 (2)	0.0352 (6)	
O10	0.0928 (3)	−0.3418 (2)	0.3282 (2)	0.0483 (7)	
O11	0.3254 (3)	0.3184 (2)	0.3709 (2)	0.0412 (6)	
H11C	0.4125	0.3135	0.3739	0.062*	
H11D	0.3080	0.3810	0.3942	0.062*	
O12	0.0844 (3)	0.2909 (2)	0.0208 (2)	0.0377 (6)	
H12D	0.0086	0.2548	0.0113	0.057*	
H12E	0.0657	0.3553	0.0074	0.057*	
O13	0.2045 (3)	0.0988 (2)	0.1947 (3)	0.0557 (8)	
H13A	0.1389	0.0784	0.2229	0.084*	
H13C	0.2825	0.0788	0.2318	0.084*	
O14	0.2161 (3)	0.4906 (2)	0.4629 (2)	0.0426 (6)	
H14A	0.2556	0.5506	0.4612	0.064*	
H14B	0.1309	0.4922	0.4286	0.064*	
O15	0.1553 (3)	0.4549 (2)	0.9108 (3)	0.0534 (8)	
H15B	0.2321	0.4841	0.9461	0.080*	
H15C	0.0889	0.4979	0.9122	0.080*	

### Atomic displacement parameters (Å^2^)

**Table:**

	*U*^11^	*U*^22^	*U*^33^	*U*^12^	*U*^13^	*U*^23^
Cu1	0.0256 (2)	0.0291 (2)	0.0397 (3)	0.00437 (17)	0.01238 (18)	0.00152 (17)
Cu2	0.0280 (2)	0.0263 (2)	0.0380 (3)	0.00544 (17)	0.01270 (18)	0.00209 (17)
Cd1	0.02616 (17)	0.03008 (17)	0.03877 (18)	0.00474 (11)	0.01123 (11)	−0.00016 (11)
C1	0.0277 (17)	0.0302 (17)	0.0338 (18)	0.0049 (14)	0.0037 (14)	−0.0031 (14)
C2	0.0284 (18)	0.0325 (18)	0.044 (2)	0.0067 (14)	0.0072 (15)	0.0018 (15)
C3	0.0324 (19)	0.0315 (18)	0.042 (2)	0.0008 (15)	−0.0007 (16)	0.0022 (15)
C4	0.043 (2)	0.0287 (18)	0.041 (2)	0.0074 (16)	−0.0050 (17)	−0.0007 (15)
C5	0.0335 (19)	0.0363 (19)	0.040 (2)	0.0089 (15)	0.0029 (16)	−0.0068 (15)
C6	0.0276 (17)	0.0320 (18)	0.0331 (18)	0.0035 (14)	0.0032 (14)	−0.0040 (14)
C12	0.064 (3)	0.036 (2)	0.059 (3)	0.004 (2)	0.006 (2)	0.0156 (19)
C7	0.0263 (17)	0.039 (2)	0.0357 (19)	0.0067 (14)	0.0072 (14)	−0.0068 (15)
C8	0.0267 (18)	0.044 (2)	0.041 (2)	0.0026 (15)	0.0142 (15)	−0.0037 (16)
C9	0.0232 (17)	0.041 (2)	0.0287 (17)	0.0014 (14)	0.0068 (13)	0.0010 (14)
C10	0.0295 (18)	0.0327 (18)	0.044 (2)	0.0005 (15)	0.0091 (15)	0.0044 (15)
C11	0.0260 (18)	0.0343 (19)	0.0328 (18)	−0.0010 (15)	0.0021 (14)	−0.0006 (14)
C13	0.0312 (18)	0.0292 (17)	0.0288 (17)	0.0023 (14)	0.0021 (14)	0.0033 (13)
C18	0.0325 (19)	0.0351 (19)	0.0301 (18)	−0.0005 (15)	0.0020 (14)	0.0003 (14)
C17	0.042 (2)	0.036 (2)	0.0361 (19)	−0.0030 (16)	0.0065 (16)	0.0047 (15)
C16	0.047 (2)	0.0324 (19)	0.040 (2)	−0.0014 (16)	−0.0001 (17)	0.0103 (15)
C15	0.041 (2)	0.0307 (18)	0.0338 (19)	0.0055 (15)	−0.0017 (16)	−0.0005 (14)
C14	0.0355 (19)	0.0317 (18)	0.0347 (18)	0.0034 (15)	0.0049 (15)	0.0027 (14)
C24	0.047 (2)	0.034 (2)	0.065 (3)	0.0058 (17)	0.017 (2)	−0.0012 (18)
C19	0.0321 (19)	0.038 (2)	0.0352 (19)	−0.0047 (15)	0.0110 (15)	0.0008 (15)
C20	0.0291 (19)	0.040 (2)	0.046 (2)	0.0027 (15)	0.0148 (16)	0.0004 (16)
C21	0.0223 (17)	0.0376 (19)	0.0341 (18)	0.0021 (14)	0.0051 (14)	−0.0071 (14)
C22	0.0350 (19)	0.0316 (18)	0.043 (2)	0.0064 (15)	0.0115 (16)	0.0000 (15)
C23	0.0285 (17)	0.0270 (17)	0.0263 (16)	0.0073 (14)	0.0022 (13)	−0.0016 (13)
N1	0.0251 (15)	0.0350 (16)	0.0362 (16)	0.0046 (12)	0.0101 (12)	−0.0006 (12)
N2	0.0273 (15)	0.0343 (16)	0.0435 (17)	0.0026 (12)	0.0125 (13)	0.0047 (13)
N3	0.0316 (16)	0.0325 (16)	0.0398 (16)	0.0005 (12)	0.0136 (13)	−0.0008 (12)
N4	0.0304 (16)	0.0314 (15)	0.0434 (17)	0.0078 (12)	0.0140 (13)	0.0022 (12)
O1	0.0319 (13)	0.0284 (13)	0.0490 (15)	0.0075 (10)	0.0160 (11)	0.0050 (11)
O2	0.0309 (14)	0.0480 (16)	0.0585 (17)	−0.0004 (12)	0.0160 (13)	0.0110 (13)
O3	0.0306 (13)	0.0312 (13)	0.0480 (15)	0.0059 (10)	0.0198 (11)	0.0047 (11)
O4	0.0326 (14)	0.0335 (14)	0.0608 (18)	0.0093 (11)	0.0144 (12)	0.0000 (12)
O5	0.0508 (17)	0.0351 (14)	0.0559 (17)	0.0065 (12)	0.0130 (14)	0.0120 (12)
O6	0.0366 (14)	0.0241 (12)	0.0479 (15)	0.0053 (10)	0.0172 (11)	0.0054 (10)
O7	0.0290 (14)	0.0411 (15)	0.0608 (17)	0.0061 (11)	0.0156 (12)	−0.0093 (13)
O8	0.0304 (13)	0.0287 (12)	0.0420 (14)	0.0065 (10)	0.0141 (11)	0.0045 (10)
O9	0.0332 (14)	0.0265 (12)	0.0469 (15)	0.0008 (10)	0.0112 (11)	0.0030 (10)
O10	0.0637 (19)	0.0301 (14)	0.0515 (17)	0.0137 (13)	0.0121 (14)	0.0079 (12)
O11	0.0296 (13)	0.0465 (15)	0.0452 (15)	0.0062 (11)	0.0071 (11)	−0.0082 (12)
O12	0.0313 (13)	0.0406 (14)	0.0419 (14)	0.0023 (11)	0.0088 (11)	0.0053 (11)
O13	0.0466 (17)	0.0364 (15)	0.093 (2)	0.0109 (13)	0.0331 (17)	0.0098 (15)
O14	0.0462 (16)	0.0375 (14)	0.0413 (15)	0.0083 (12)	0.0054 (12)	−0.0002 (11)
O15	0.0424 (17)	0.0470 (17)	0.070 (2)	0.0006 (13)	0.0068 (15)	0.0164 (15)

### Geometric parameters (Å, °)

**Table:**

Cu1—O1	1.887 (3)	C11—O4	1.224 (5)
Cu1—N2	1.889 (3)	C11—O3	1.296 (4)
Cu1—N1	1.916 (3)	C13—O6	1.315 (4)
Cu1—O3	1.960 (2)	C13—C14	1.411 (5)
Cu2—O6	1.882 (2)	C13—C18	1.427 (5)
Cu2—N4	1.898 (3)	C18—C17	1.402 (5)
Cu2—N3	1.922 (3)	C18—C19	1.431 (5)
Cu2—O8	1.985 (2)	C17—C16	1.370 (6)
Cd1—O11	2.258 (3)	C17—H17	0.9300
Cd1—O12	2.260 (3)	C16—C15	1.395 (6)
Cd1—O3	2.288 (2)	C16—H16	0.9300
Cd1—O13	2.345 (3)	C15—O10	1.364 (5)
Cd1—O8	2.380 (2)	C15—C14	1.372 (5)
Cd1—O9	2.432 (3)	C14—H18	0.9300
Cd1—O4	2.545 (3)	C24—O10	1.425 (5)
C1—O1	1.320 (4)	C24—H24A	0.9600
C1—C2	1.396 (5)	C24—H24B	0.9600
C1—C6	1.431 (5)	C24—H24C	0.9600
C2—C3	1.387 (5)	C19—N3	1.282 (5)
C2—H2	0.9300	C19—H19	0.9300
C3—O5	1.366 (5)	C20—N3	1.459 (5)
C3—C4	1.391 (6)	C20—C21	1.522 (5)
C4—C5	1.359 (6)	C20—H20A	0.9700
C4—H4	0.9300	C20—H20B	0.9700
C5—C6	1.399 (5)	C21—O7	1.260 (4)
C5—H5	0.9300	C21—N4	1.296 (5)
C6—C7	1.434 (5)	C22—N4	1.440 (5)
C12—O5	1.433 (5)	C22—C23	1.501 (5)
C12—H12A	0.9600	C22—H22A	0.9700
C12—H12B	0.9600	C22—H22B	0.9700
C12—H12C	0.9600	C23—O9	1.230 (4)
C7—N1	1.281 (5)	C23—O8	1.296 (4)
C7—H7	0.9300	O11—H11C	0.8502
C8—N1	1.464 (5)	O11—H11D	0.8500
C8—C9	1.520 (5)	O12—H12D	0.8499
C8—H8A	0.9700	O12—H12E	0.8500
C8—H8B	0.9700	O13—H13A	0.8499
C9—O2	1.249 (5)	O13—H13C	0.8500
C9—N2	1.302 (5)	O14—H14A	0.8500
C10—N2	1.450 (5)	O14—H14B	0.8500
C10—C11	1.515 (5)	O15—H15B	0.8500
C10—H10A	0.9700	O15—H15C	0.8499
C10—H10B	0.9700		
			
O1—Cu1—N2	175.76 (13)	O6—C13—C18	125.0 (3)
O1—Cu1—N1	96.85 (12)	C14—C13—C18	117.3 (3)
N2—Cu1—N1	84.08 (13)	C17—C18—C13	118.9 (3)
O1—Cu1—O3	95.95 (11)	C17—C18—C19	117.1 (3)
N2—Cu1—O3	83.07 (12)	C13—C18—C19	123.9 (3)
N1—Cu1—O3	167.14 (12)	C16—C17—C18	122.9 (4)
O6—Cu2—N4	177.36 (13)	C16—C17—H17	118.6
O6—Cu2—N3	97.34 (12)	C18—C17—H17	118.6
N4—Cu2—N3	83.47 (13)	C17—C16—C15	117.7 (3)
O6—Cu2—O8	96.54 (11)	C17—C16—H16	121.1
N4—Cu2—O8	82.68 (12)	C15—C16—H16	121.1
N3—Cu2—O8	166.11 (12)	O10—C15—C14	123.4 (4)
O11—Cd1—O12	167.94 (10)	O10—C15—C16	114.9 (3)
O11—Cd1—O3	90.74 (10)	C14—C15—C16	121.7 (4)
O12—Cd1—O3	91.08 (9)	C15—C14—C13	121.3 (4)
O11—Cd1—O13	96.65 (11)	C15—C14—H18	119.4
O12—Cd1—O13	95.42 (11)	C13—C14—H18	119.4
O3—Cd1—O13	81.39 (10)	O10—C24—H24A	109.5
O11—Cd1—O8	88.27 (9)	O10—C24—H24B	109.5
O12—Cd1—O8	94.42 (9)	H24A—C24—H24B	109.5
O3—Cd1—O8	158.18 (9)	O10—C24—H24C	109.5
O13—Cd1—O8	77.08 (10)	H24A—C24—H24C	109.5
O11—Cd1—O9	86.73 (9)	H24B—C24—H24C	109.5
O12—Cd1—O9	85.32 (9)	N3—C19—C18	125.7 (3)
O3—Cd1—O9	147.44 (9)	N3—C19—H19	117.2
O13—Cd1—O9	131.15 (10)	C18—C19—H19	117.2
O8—Cd1—O9	54.25 (8)	N3—C20—C21	109.8 (3)
O11—Cd1—O4	91.13 (10)	N3—C20—H20A	109.7
O12—Cd1—O4	80.39 (9)	C21—C20—H20A	109.7
O3—Cd1—O4	53.67 (9)	N3—C20—H20B	109.7
O13—Cd1—O4	134.52 (10)	C21—C20—H20B	109.7
O8—Cd1—O4	148.13 (8)	H20A—C20—H20B	108.2
O9—Cd1—O4	93.90 (8)	O7—C21—N4	127.0 (4)
O1—C1—C2	118.2 (3)	O7—C21—C20	119.4 (3)
O1—C1—C6	124.2 (3)	N4—C21—C20	113.6 (3)
C2—C1—C6	117.6 (3)	N4—C22—C23	108.3 (3)
C3—C2—C1	121.4 (4)	N4—C22—H22A	110.0
C3—C2—H2	119.3	C23—C22—H22A	110.0
C1—C2—H2	119.3	N4—C22—H22B	110.0
O5—C3—C2	114.5 (4)	C23—C22—H22B	110.0
O5—C3—C4	124.5 (3)	H22A—C22—H22B	108.4
C2—C3—C4	121.0 (4)	O9—C23—O8	120.6 (3)
C5—C4—C3	117.9 (4)	O9—C23—C22	121.7 (3)
C5—C4—H4	121.1	O8—C23—C22	117.7 (3)
C3—C4—H4	121.1	C7—N1—C8	122.0 (3)
C4—C5—C6	123.6 (4)	C7—N1—Cu1	124.7 (3)
C4—C5—H5	118.2	C8—N1—Cu1	113.2 (2)
C6—C5—H5	118.2	C9—N2—C10	124.0 (3)
C5—C6—C1	118.3 (3)	C9—N2—Cu1	118.8 (3)
C5—C6—C7	117.1 (3)	C10—N2—Cu1	117.0 (2)
C1—C6—C7	124.3 (3)	C19—N3—C20	122.9 (3)
O5—C12—H12A	109.5	C19—N3—Cu2	123.0 (3)
O5—C12—H12B	109.5	C20—N3—Cu2	113.8 (2)
H12A—C12—H12B	109.5	C21—N4—C22	124.0 (3)
O5—C12—H12C	109.5	C21—N4—Cu2	119.2 (3)
H12A—C12—H12C	109.5	C22—N4—Cu2	116.8 (2)
H12B—C12—H12C	109.5	C1—O1—Cu1	124.3 (2)
N1—C7—C6	124.8 (3)	C11—O3—Cu1	115.3 (2)
N1—C7—H7	117.6	C11—O3—Cd1	97.6 (2)
C6—C7—H7	117.6	Cu1—O3—Cd1	146.97 (13)
N1—C8—C9	110.0 (3)	C11—O4—Cd1	87.5 (2)
N1—C8—H8A	109.7	C3—O5—C12	117.5 (3)
C9—C8—H8A	109.7	C13—O6—Cu2	123.8 (2)
N1—C8—H8B	109.7	C23—O8—Cu2	114.2 (2)
C9—C8—H8B	109.7	C23—O8—Cd1	92.9 (2)
H8A—C8—H8B	108.2	Cu2—O8—Cd1	151.75 (12)
O2—C9—N2	127.3 (4)	C23—O9—Cd1	92.2 (2)
O2—C9—C8	119.4 (3)	C15—O10—C24	117.8 (3)
N2—C9—C8	113.2 (3)	Cd1—O11—H11C	109.4
N2—C10—C11	107.5 (3)	Cd1—O11—H11D	109.4
N2—C10—H10A	110.2	H11C—O11—H11D	109.5
C11—C10—H10A	110.2	Cd1—O12—H12D	109.3
N2—C10—H10B	110.2	Cd1—O12—H12E	109.4
C11—C10—H10B	110.2	H12D—O12—H12E	109.5
H10A—C10—H10B	108.5	Cd1—O13—H13A	109.1
O4—C11—O3	121.0 (3)	Cd1—O13—H13C	109.4
O4—C11—C10	121.9 (3)	H13A—O13—H13C	109.5
O3—C11—C10	117.1 (3)	H14A—O14—H14B	109.5
O6—C13—C14	117.7 (3)	H15B—O15—H15C	109.5

### Hydrogen-bond geometry (Å, °)

**Table:**

*D*—H···*A*	*D*—H	H···*A*	*D*···*A*	*D*—H···*A*
O11—H11D···O14	0.85	1.91	2.715 (4)	158
O13—H13A···O6	0.85	2.14	2.987 (4)	175
O13—H13C···O1	0.85	2.21	2.829 (4)	129
O14—H14B···O9	0.85	2.48	3.045 (4)	125
O11—H11C···O7^i^	0.85	1.91	2.722 (4)	159
O12—H12D···O2^ii^	0.85	2.11	2.667 (4)	123
O12—H12E···O15^iii^	0.85	2.13	2.737 (4)	129
O14—H14A···O7^iv^	0.85	2.17	2.791 (4)	130
O15—H15B···O4^v^	0.85	2.55	3.067 (4)	121
O15—H15C···O9^iv^	0.85	2.36	2.934 (4)	126

Symmetry codes: (i) *x*+1, *y*, *z*; (ii) *x*−1, *y*, *z*; (iii) *x*, *y*, *z*−1; (iv) −*x*, −*y*+1, −*z*+1; (v) *x*, *y*, *z*+1.
